# Wnt/*β*‐catenin signalling controls bile duct regeneration by regulating differentiation of ductular reaction cells

**DOI:** 10.1111/jcmm.16017

**Published:** 2020-10-30

**Authors:** Nan Wang, Rui Kong, Wei Han, Jie Lu

**Affiliations:** ^1^ Department of Gastroenterology Shanghai Tenth People's Hospital Affiliated to Tongji University Tongji University School of Medicine Shanghai China

**Keywords:** bile duct regeneration, ductular reaction cells, Lgr5, Wnt, *β*‐catenin signalling

## Abstract

Recently, the incidence of bile duct‐related diseases continues to increase, and there is no effective drug treatment except liver transplantation. However, due to the limited liver source and expensive donations, clinical application is often limited. Although current studies have shown that ductular reaction cells (DRCs) reside in the vicinity of peribiliary glands can differentiate into cholangiocytes and would be an effective alternative to liver transplantation, the role and mechanism of DRCs in cholangiole physiology and bile duct injury remain unclear. A 3,5‐diethoxycarbonyl‐1,4‐dihydrocollidine (DDC)‐enriched diet was used to stimulate DRCs proliferation. Our research suggests DRCs are a type of intermediate stem cells with proliferative potential that exist in the bile duct injury. Meanwhile, DRCs have bidirectional differentiation potential, which can differentiate into hepatocytes and cholangiocytes. Furthermore, we found DRCs highly express Lgr5, and Lgr5 is a molecular marker for neonatal DRCs (*P* < .05). Finally, we confirmed Wnt/β‐catenin signalling achieves bile duct regeneration by regulating the expression of Lgr5 genes in DRCs (*P* < .05). We described the regenerative potential of DRCs and reveal opportunities and source for the treatment of cholestatic liver diseases.

## 1 INTRODUCTION

For a long time, people's understanding of the bile duct is mainly considered as the excretory duct of bile, but little is known about its characteristics. The bile duct system is divided into intrahepatic and extrahepatic bile ducts, the intrahepatic biliary system acting as a liver bracket plays an important role in the process of liver repair, and the proliferation of bile duct cells directly regulates the radius of the bile duct.^1^

Biliary epithelial cells in extrahepatic large bile duct and intrahepatic small bile duct are not homogeneous, showing obvious heterogeneity in morphology, biochemistry and function.^2^ The bile duct located outside the liver belongs to the differentiated mature cells, while the small intrahepatic bile duct belongs to a class of immature cells.^3^ It is generally believed that cholangiocytes in small bile ducts have proliferative capacity and show functional plasticity in diseases.^4^ Without the regeneration of the bile duct tree structure and the repair of the function of the bile duct epithelial cells, liver regeneration would be an ineffective regeneration.^5^ Only have hepatocyte regeneration but poor bile duct regeneration, the hepatic plate cannot be arranged in an orderly manner around the bile duct, and the bile secreted by the hepatocytes cannot flow into the bile duct system.^6^ Toxic substances in the liver retention, causing hepatocyte membrane and cellular ultrastructural changes, cell degeneration, necrosis and ultimately cirrhosis. Therefore, studying the regeneration of bile duct cells is the key to the treatment of bile duct injury‐related diseases.^7^

Ductular reaction (DR) is a common injury response in lots of hepatobiliary diseases and is characterized by the proliferation of DRCs.^8^ DR in humans is an intriguing pathologic feature associated with fibrosis and bile duct hyperplasia.^8^ Meanwhile, DRCs gradually attain the complete epithelial phenotype of hepatocytes or cholangiocytes during differentiation.^9^ Although DR has attracted the attention of scientists for its relevance to differentiation of hepatocytes and its important role in promoting liver regeneration, the mechanism of its regulation of this process has not been fully understood.^10^

In the previous work, we were surprised to find a class of intermediate cells resemble progenitor cell in DDC induced bile duct injury mice model, which were defined as abnormal hyperplasia of DRCs. These cells can differentiate into hepatocytes and bile duct cells, which are most likely important precursors of nascent bile duct cells. We thought it will be a new way for future treatment of biliary tract‐related diseases to clarify the source of new cholangiocytes and carry out cell transplantation technology.

We separated DRCs from mice fed with DDC‐enriched diet, a robust model of bile duct injury and biliary fibrosis, suggesting a good platform for isolating DRCs.^11^

In DDC mice model, we found DRCs proliferate at the capillary bile duct and move radially from the portal area to the hepatic parenchymal region. Some DRCs further differentiate into mature bile duct cells. This process is an important stage to achieve structural reconstruction of damaged bile duct tree and repair of liver tissue function. It can be seen that the effective coordination between pathological amplification and normal maturation of DRCs plays a crucial role in bile duct cells regeneration, and the study of its regulatory mechanism has also become a hot topic, also, it is the key to the treatment of bile duct injury‐related diseases.

We were more surprised to find Leucine‐rich repeat‐containing G protein‐coupled receptor 5 (Lgr5), a stem cell marker that is rarely expressed in general, showed enhanced expression in neonatal DRCs. Lgr5 also known as G protein‐coupled receptor 49/67 (Gpr49 or Gpr67) is a protein that in humans is encoded by the Lgr5 gene.^12^ It is a member of GPCR class A receptor proteins, R‐spondins (Rspos) are the biological ligands of Lgr5.^13^ Lgr5 as a well‐established stem cell marker in certain types of tissue such as in the muscle, placenta, spinal cord and brain.^14^ This wholly due to the fact that they are enriched in truly multipotent stem cells compared to their immediate progeny, the transit‐amplifying cells.^15^ Lgr5 is also transcriptional target of Wnt/*β*‐catenin signalling, although its ligand remains elusive, it has been shown that costimulation with Rspos 1 and Wnt‐3a induces increased internalization of Lgr5.^16^ Binding of Rspos to Lgr5 isolates Rnf43/Znrf3, which stabilized the Wnt receptor complex on the cell surface and simultaneously amplified the canonical *β*‐catenin‐dependent Wnt signalling.^17^ The central mediator of typical Wnt signalling is *β*‐catenin, which acts as a gene transcription activator by combining with nuclear complex T‐cell factor/lymphoid enhancer factor (TCF/LEF)..^18^, ^19^After the Wnt ligand binds to the cell membrane, *β*‐catenin accumulates in the cytoplasm and transfers to the nucleus to initiate transcription of the target gene.^20^

Increasing evidence supports the important role of β‐catenin in regulating hepatocyte proliferation during development and regeneration. In the study of liver culture in vitro, Wnt‐3a promoted the differentiation of hepatocytes into biliary epithelial cells (BECs),^21^, ^22^ while *β*‐catenin with Apc deletion in mouse hepatocytes stably induced premature differentiation into BECs.^23^ In the adult liver, Wnt pathway is only active in hepatocytes surrounding the central vein,^24^ whereas in the bile duct, Wnt signalling becomes active after liver injury.^25^ These studies support Wnt/*β*‐catenin signal to play an active role in biliary tract induction. However, so far, the role of Wnt signalling in the repair and regeneration of bile duct injury is still unclear. Therefore, we speculated Lgr5 may be a specific marker for such intermediate cells, and the differentiation process of DRCs is regulated by Wnt signalling pathway.

In this study, we applied a Lgr5 CreERT2 DDC mouse model that enables us to identify and study DRCs. We have utilized various anatomic and immunohistochemical features including CK19 and CK7 as surface markers of cholangiocyte development,^26^ AFP as a surface marker for new hepatocytes^27^ and epithelial cell adhesion molecule (EpCAM), a surface marker of hepatic progenitor cell.^28^ In addition, we study the interrelationship of the proliferative DRCs and cholangiocytes during biliary injury to assess the contribution of DRCs during in mice models of healthy biliary homeostasis and cholangiole regeneration.

This study not only extends our understanding of the Wnt/*β*‐catenin signalling pathway and its regulatory mechanism involved in intrahepatic biliary regeneration through DRCs proliferation, but also provides novel therapeutic targets in hepatobiliary disease for further study.

## 2 MATERIALS AND METHODS

### 2.1 Mice and treatment

The Lgr5‐CreERT2 (8‐week‐old, male) mouse strain was purchased from the Model Animal Research Center of Nanjing University. All mice were kept under barrier conditions and were raised in our laboratory. After all mice were adapted to normal diet and drinking for one week, set up normal diet control group and DDC diet experimental group. The DDC model mice were fed with 1% DDC (Sigma‐Aldrich) for 4 weeks and it mainly caused bile duct injury and intrahepatic cholestasis. After the modelling was completed, the mice in each group were anesthetized, shaved, and disinfected. The abdominal cavity was opened, and the mice were quickly dissected. After removing the whole liver, they were rinsed with physiological saline. Fresh livers of some mice were stored in 4% paraformaldehyde overnight at 4°C for paraffin sectioning. The remaining livers were cut and put into liquid nitrogen and stored at −80°C in ultra‐low temperature refrigerator. All animal experiments approved by the Animal Care and Use Committee of Tongji University and were performed in accordance with the National Institutes of Health Guidelines.

### 2.2 Histopathology

A portion of the live tissue was fixed in 4% paraformaldehyde for at least 24 hour and then was subjected to dehydration and penetration. The specimen was then embedded in paraffin. The section was cut at a thickness of 4 μm for haematoxylin and eosin (H&E) staining and Masson's trichrome staining. Pathological changes were observed by light microscopy.

### 2.3 Immunohistochemistry

The prepared sections (4 μm) were heated at 60°C for 1 hour and then rehydrated with xylene and different concentrations of alcohol. The sections were washed three times with phosphate buffer solution (PBS) to block non‐specific binding sites, immersed in 5% bovine serum albumin (BSA) for 20 minutes at 37°C and then incubated for 10 minutes. Specimens were incubated overnight at 4°C with primary antibodies including Ck7 (1:200), Ck19 (1:200), AFP (1:500) and Lgr5 (1:100). On the second day, after incubation with a appropriate secondary antibody for 30 minutes at 37°C, the sections were analysed with a diaminobenzidine (DAB) kit to detect antibody binding. Finally, the slices were observed by optical microscopy.

### 2.4 Microfil™ injection

The mice in the normal diet group and the DDC diet experimental group at the 4th week were selected, and the mice were sacrificed by intravenous injection of excess ether. We visualized the common bile duct (CHD), tied a suture around it and injected it with 1 mL of PBS with a small needle. Microfil™ was subsequently injected into the CHD until the IHBDs were filled with dye. The clearing protocol was performed per the Flow tech website with serial ethanol and methyl salicylate.

### 2.5 DRCs isolation

After the whole livers were dissected, finely diced, they were treated with dispose (1000 μL/mL; Godo Shusei Co. Ltd.) in 2‐[4‐(hydroxyethyl)‐1‐piperazinyl] ethane sulphonic acid (Sigma Chemical Co.) and buffered DMEM (Gibco) for 60 minutes at 37°C.^29^, ^30^ The liver specimens were then dispersed into single cells by pipetting and filtered through a nylon mesh filter with a pore size of 132 μm (Nihon Rikagaku Kikai Co., Ltd) and the undigested clot on the mesh was recovered and redigested. Magnetic M‐450 Dynabeads covalently coated with a combination of sheep anti‐mouse immunoglobulin G (IgG) and an antibody against rabbit EpCAM (Santa Cruz Biotechnology) were used to separate the mice ductular cells, as previously described.^31^, ^32^ EpCAM‐positive cells were isolated using a separation magnet (BD Imagnet, BD Biosciences) according to the manufacturer's instructions. The supernatant is the negative fraction. The beads were precoated by incubation in TBS containing 10 mmol/L CaCl2 and 1% bovine serum albumin (BSA) to prevent non‐specific binding to the cells. Isolated DRCs viability was determined with Trypan blue exclusion, which exceeded 95%.

### 2.6 Cell culture and cell proliferation analysis

The isolated DRCs were cultured in DMEM culture medium (Gibco Industries) supplemented with 10% foetal bovine serum (Hyclone), 100 U/mL penicillin and 100 g/mL streptomycin (Gibco) in a humidified incubator at 37°C with 5% CO_2_.

The cells were plated in 96‐well plates, and 10 µL CCK‐8 solution (Peptide Institute) was then added to each well. The plate was maintained in a dark incubator (37°C, 5% CO_2_) for 2 hours Except for Normal Control group, other group was treated with β‐catenin overexpression plasmid, empty plasmid and inhibitor shRNA, respectively. Cell viability was measured with the CCK‐8 assay at a wavelength of 450 nm by using a microplate reader for further half‐maximal inhibitory concentration (IC50) analysis.

The DRCs were divided into four groups:

1. Control group: no treatment;
2. EV group: treated with empty plasmid;
3. *β*‐catenin group: treated with *β*‐catenin overexpression plasmid;
4. shRNA group: treated with inhibitor shRNA.

### 2.7 Immunofluorescence

Fresh liver tissues were collected after euthanasia of the mice and then fixed in 4% paraformaldehyde (PFA, Sigma) for 1 hour at 4°C. The tissues were washed with PBS three times, then the tissues were dehydrated overnight in 30% sucrose (dissolved in PBS) at 4°C. The tissues were embedded in OCT (Sakura) for 2 hours on day 2 and stored at −80°C. 5 µm frozen sections were collected on slides. Before immunofluorescent staining, the tissue sections were dried at room temperature for 5 minutes and then put in PBS to dissolve OCT for 5 minutes. Next, tissue sections were ruptured with 5% PBST (containing 5% donkey serum in PBS and 0.1% Triton X‐100 in PBS) for 30 minutes at room temperature. The non‐specific antigen‐binding sites were blocked with 5% BSA, and the sections were then incubated with primary antibodies at 4°C overnight in dark. The slides incubated with secondary antibodies for 30 minutes at room temperature in dark on day 2 and then were incubated with 2‐(4‐amidinophenyl)‐6‐indolecarbamidine dihydrochloride (DAPI) (Vector Laboratories). Olympus confocal (FV1200) was used to observed immunostaining images. Images were analysed by ImageJ (NIH) software.

### 2.8 SYBR green real‐time PCR

The TRIzol reagent was used to extract total RNA. cDNA was synthesized using SuperScript II reverse transcriptase with Oligo (dT; Invitrogen). Real‐time PCR was carried out with a 7500 real‐time PCR system (Applied Biosystems) using the SYBR Green PCR Kit (Takara Japan) according to manufacturers’ instructions. The levels of the target genes were normalized to *β*‐actin. The primer pairs used in our experiment are listed in Table 1.

**Table 1:** The primers used in experiments

Gene		Primer sequence(5′‐3′)
*β*‐actin	Forward	GGCTGTATTCCCCTCCATCG
	Reverse	CCAGTTGGTAACAATGCCATGT
Lgr5	Forward	CACCCCAATGCGTTTTCTAC
	Reverse	GATGGTATCAGGCTCTGTAAGG
Ck7	Forward	TTCGCTCAGAAGATCAGCAGA
	Reverse	ACGGGTGAGTGACGAGGTAAT
Ck19	Forward	GTTCAGTACGCATTGGGTCAG
	Reverse	GAGGACGAGGTCACGAAGC
AFP	Forward	AGCTTCCACGTTAGATTCCTCC
	Reverse	ACAAACTGGGTAAAGGTGATGG

### 2.9 Western blotting

Total cellular proteins were extracted using radioimmunoprecipitation assay (RIPA) buffer (Kaiji Biology) containing protease inhibitors, and the protein concentration was detected with a BCA kit (Kaiji Biology). Equal amounts of protein samples (20 µg) were resolved by SDS‐polyacrylamide gel electrophoresis and transferred to polyvinyl difluoride (PVDF) membranes (Millipore Corp). The membranes were sequentially blocked with 5% BSA dissolved in phosphate buffered saline containing 0.1% Tween‐20 (PBST) to block non‐specific binding sites for 1 hour, followed by incubation in monoclonal primary antibodies overnight at 4°C. The primary antibodies were diluted as follows: *β*‐actin (1:1000), Ck7 (1:200), Ck19 (1:200), AFP (1:500), Lgr5 (1:100), *β*‐actin was used as an internal control. On the second day, the membranes were washed with PBST three times and then incubated with the secondary antibody horseradish peroxidase‐conjugated anti‐rabbit or anti‐mouse IgG (1:2000) for 1 hour at 37°C. After three washes, the membranes were scanned using the Odyssey two‐colour infrared laser imaging system (Licor).

### 2.10 Statistical analysis

All data were collected from at least three independent experiments as indicated. GraphPad Prism Software version 6.0 for Windows (GraphPad) was used for all statistical analysis. All data are presented as mean values ± SEM. For compare data, data significance was analysed using a two‐tailed unpaired Student's *t* test. A one‐way ANOVA was used when more than two groups were being compared, a two‐way ANOVA was used when two groups were split between two independent variables, and a two‐tailed Mann‐Whitney *U* test was used when n was too small to determine normal distribution or the data were non‐parametric. F tests were used to compare variances between groups. Values of *P < .05, **P < .01 and ***P < .001 were considered statistically significant.

## 3 RESULTS

### 3.1 DDC feeding increases mouse DRCs

**Figure 1:**
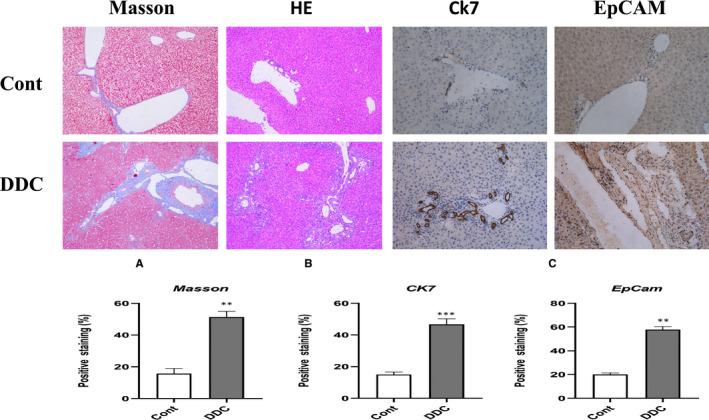
DDC feeding causes bile duct injury and Ductular Reactive Cells. A and B, Representative H&E and Masson stained biliary images of two groups. The DDC diet mouse display bold tube thickening, peripheral fibrosis, cholestasis and small bile duct regeneration. C, Immunohistochemical staining showed the positive area of CK7 and EpCAM (Original magnification, ×200). The results showed statistically significant differences among the different groups

We observed the pathological changes of liver tissue through HE staining, it was found that compared with the normal diet group, the DDC model group showed obvious liver fibrosis, extracellular matrix deposition, cholestasis and proliferation of the small original tube‐like structure around the bile duct (Figure 1A). We re‐examined the extent of bile duct fibrosis by Masson's staining, and the results confirmed that there was significant collagen deposition around the portal vein in the DDC model group, while the normal diet group had no obvious pathological changes (Figure 1B).

These new primitive tube‐like structures are very similar to the biological shape of the bile duct. We further detected by immunohistochemical staining and found that compared with the normal diet group, cholangiocytes surface markers CK7 and hepatic progenitor cells surface marker EpCAM in the DDC diet model group increased significantly, proving the existence of regeneration of neonatal bile duct cells, suggesting that these original tube‐like structures are new small bile ducts, which are composed of DRCs (Figure 1C).

### 3.2 DRCs have bidirectional differentiation potential and express high levels of stem cells markers Lgr5

**Figure 2:**
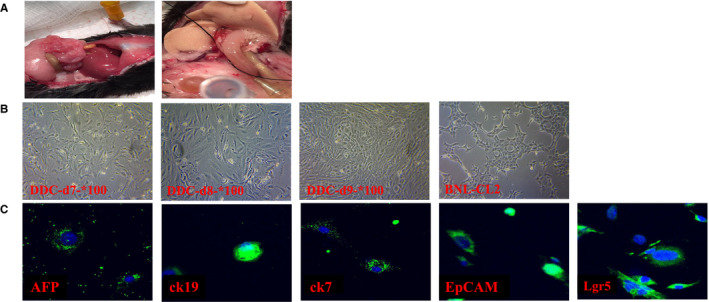
Ductular reaction cells (DRCs) isolation and culture in vitro. A, DRCs cells were isolated from the DDC mouse model and (B) further cultured in vitro (Original magnification, 100×). C, Isolated DRCs express hepatocyte marker (AFP), cholangiocyte markers (CK7, CK19) and the stem cell marker (Lgr5, EpCAM) as detected with immunofluorescence

To further study the biological characteristics of regenerated DRCs, we isolated it from DDC diet model (4 weeks) by two‐step collagenase lavage method, immunomagnetic bead method and density gradient centrifugation (Figure 2A). After cultivating neoformative DRCs for a week, it was found that the morphology and growth characteristics of these cells were very similar to embryonic liver stem cells (BNL‐Cl2) (Figure 2B). Immunofluorescence staining showed that such DRCs expressed CK19 and CK7, simultaneously expressed AFP, EpCAM and Lgr5 (Figure 2C), suggesting that DRCs can further differentiate into cholangiocytes and hepatocytes, indicating that it is a class of cells with full bidirectional differentiation potential.

### 3.3 Lgr5 is a special molecular marker for nascent DRCs

**Figure 3:**
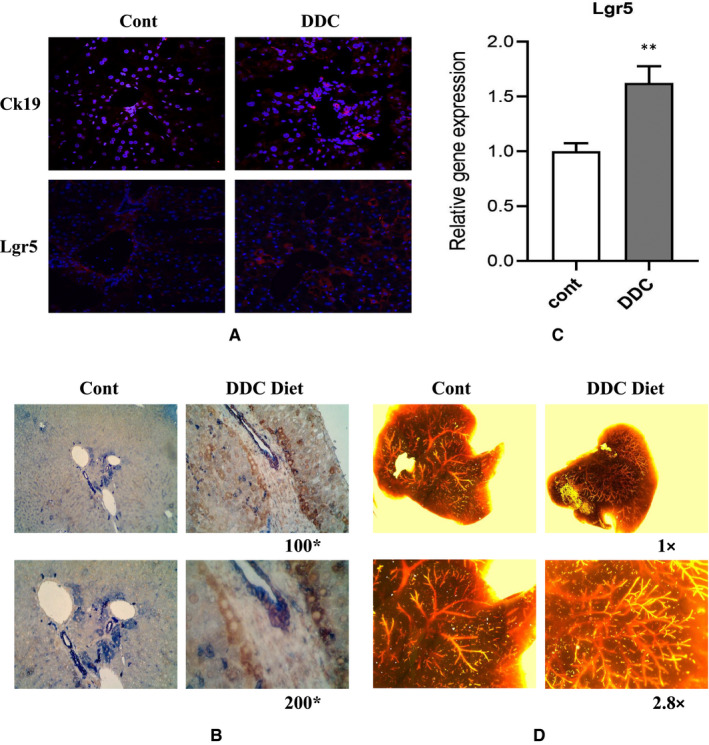
Lgr5 increased expression in DRCs and DRCs contribute to biliary regeneration in mice treated with DDC diet. A, Representative immunofluorescent staining for Lgr5 (red) and CK19 (red) showed that in the DDC diet group, lgr5+ cells increased significantly and can give birth to cholangiocytes. B, Representative immunohistochemical co‐staining images showing the expression of Lgr5 (brown) and Ck7 (blue) in DRCs and Cont groups (Magnification ×100 and ×200). C, For freshly isolated DRCs in DDC group and non‐DRCs derived from Control group (paired mice), gene expression quantified by RT‐PCR showed that DRCs expressed relatively higher levels of stem cell markers Lgr5, compared to the control. (**P < .01). D, In the control model, the biliary was normal after Microfil injection. In the DDC diet (4 wk), the intrahepatic biliary network filled with Microfil was markedly proliferative (Magnification 1× and 2.8×)

Observation by immunofluorescence confocal microscope showed that compared with the normal control mice, Lgr5+ cells were significantly increased around the small nascent bile duct in the DDC model mice, and the surface marker of cholangiocytes Ck19 was also significantly increased, indicating that it could differentiate into mature bile duct cells (Figure 3A). Further immunohistochemical co‐staining revealed that the expression of CK19 and stem cell surface marker Lgr5 around the neonatal small bile duct in the DDC model group of Lgr5ERT2 mice was significantly increased (Figure 3B). We found by RT‐PCR analysis that the expression of Lgr5+ cells in the DDC model group was significantly higher than that in the control group at the transcription level, and this was statistically different (*P* < .05) (Figure 3C). After feeding DDC and ordinary diet mice for four weeks, the structure of bile duct tree was reshaped by the Microfil technique, and it was found that the neonatal small bile duct structure of the DDC model was significantly increased compared with the ordinary control group (Figure 3D).

### 3.4 Regulatory effects of Wnt signalling on the differentiation of DRCs in vitro

**Figure 4:**
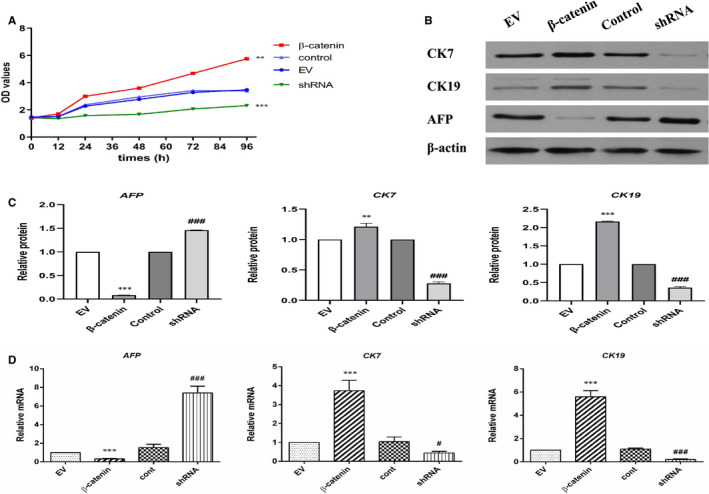
Wnt/β‐catenin signalling regulates cholangiocyte differentiation in DRCs. A, The proliferation of DRCs treated with *β*‐catenin overexpression plasmid or shRNA inhibitor was detected using CKK8 assays. B and C, The protein expression of CK7, CK19 and AFP in DRCs was detected by Western blot analysis, and the grey values were calculated. *β*‐actin was used as loading controls. D, The mRNA levels of CK7, CK19 and AFP in DRCs treated with β‐catenin overexpression plasmid or shRNA inhibitor were detected by RT‐PCR. Plotted values represent the mean ± SD. of three independent experiments (*P < .05, **P < .01, ***P < .001)

We used CCK‐8 experiment to detect the effect of *β*‐catenin overexpression on the proliferation ability of DRCs. The *β*‐catenin overexpression plasmid, empty plasmid and shRNA were transferred into DRCs respectively. By comparing the cell proliferation activity of the four groups, the experimental results suggested that after the *β*‐catenin overexpression plasmid was transferred into DRCs, compared with the empty plasmid group and the untreated negative control group, the proliferation activity of DRCs was significantly enhanced, while the transfer of shRNA significantly reduced the proliferation activity of DRCs. These above results indicate that in neonatal DRCs, the transfer of *β*‐catenin can promote the proliferation of DRCs cells, but after the transfer of shRNA, the proliferation of DRCs cells will be reduced, this difference is statistically significant (*P* < .05) (Figure 4A).

To further clarify whether Wnt/*β*‐catenin is involved in the process of bile duct injury and repair by regulating the proliferation and differentiation of DRCs, we transferred *β*‐catenin overexpression plasmid and shRNA in DRCs cultured in vitro to detect bile duct specific marker expression of CK7, CK19 and hepatic cell surface marker AFP. Through Western blot and real‐time fluorescent PCR experiments, it was found that at the protein and mRNA levels, the expression of CK7 and CK19 in DRCs was enhanced compared with the untreated control group and was significantly enhanced compared with the shRNA inhibition group. While the expression of AFP in DRCs was obviously weaker than that in untreated control group, and was significantly enhanced compared with shRNA suppression group (Figure 4B‐D). These results indicated that *β*‐catenin can up‐regulate the expression of CK7 and CK19 in DRCs, and down‐regulate the expression of AFP in vitro, suggesting that Wnt/*β*catenin signaling pathway could regulate the differentiation of DRCs.

## 4 DISCUSSION

Cholangiocytes are affected during liver injury and participate in the pathophysiology of various liver disease states. Although there is sufficient evidence that hepatocytes (the main epithelial cell type in the liver) can be regenerated, bile duct cells can also promote liver regeneration when hepatocyte regeneration is impaired.^33^, ^34^ In recent years, the morbidity and mortality of cholangiopathies are still very high, which poses a major challenge to clinical management and brings a considerable economic burden to patients and society. In‐depth research on the repair mechanism of bile duct injury is urgent. Studies have shown that DDC diet can construct a stable model of bile duct injury in mice.^35^ In this study, we successfully found nascent DRCs using the Lgr5‐CreERT2 DDC mouse model and found that some DRCs can be further differentiated into mature bile duct cells. This process is just an important stage to achieve the reconstruction of the damaged bile duct tree structure and the repair of liver tissue function.

DR is a common injury response in a variety of cholestasis and other liver diseases.^8^ Related studies have shown that DRCs are a cell group different from liver progenitor cells (HPCs) and have their unique repair function.^36^ An essential prerequisite for biliary repair is that DRCs can reconstruct the biliary structure.^37^ In this study, we further cultured the DRCs successfully isolated from the DDC mice model in vitro and found that the cell morphology and growth characteristics of these cells resemble embryonic liver stem cells, and some were able to differentiate into cholangiocytes and hepatocytes, and meanwhile, the stem cell marker Lgr5 also showed abnormally high expression in DRCs.

In DR studies, EpCAM and SOX9 are often identified as stem cell markers.^38^ CK7 or CK19 is used to identify cholangiocytes.^39^ However, cholangiocytes also express EpCAM and SOX9.^40^, ^41^ Therefore, none of these markers alone can recognize stem cell proliferation. Lgr5, a protein encoded by the Lgr5 gene in humans, was first valued as a marker for intestinal stem cells (ISCs) in 2007.^42^ Lgr5 has been shown to express on facultative stem cells responsible for tissue regeneration after injury in the liver, pancreas and stomach^43^ and cancer stem cells that promote tumour growth.^44^ Lgr5 is a molecular marker of epithelial cells, and during mice embryonic development, it is significantly increased in embryonic epithelial cells, whereas expression was extremely low in adult mice.^45^ During the process of bile duct injury induced by DDC diet, we confirmed that Lgr5 is highly expressed in neonatal DRCs. Therefore, we described Lgr5 is a special molecular marker for DRCs.

Studies have shown that many intercellular signalling pathways, such as Wnt, HGF, BMP and Notch, have been shown to be involved in the development of the liver, especially the bile duct,^46^, ^47^ of which Wnt has been proved to be of importance.^48^ In biliary system, Wnt signalling becomes active following liver injury.^25^ Furthermore, Wnt signalling was demonstrated to play an important role in regulating stem cell homeostasis by promoting the self‐renewal of stem cells and controlling differentiation in many organs.^49^ The central mediator of typical Wnt signalling is β‐catenin, and Lgr5 is an enhancer of Wnt/*β*‐catenin signalling.

Since DRCs have the potential of stem cell bidirectional differentiation and share biological functions with embryonic liver progenitor cells, these results urge us to study whether Wnt/*β*‐catenin signal is related to bile duct injury repair by regulating the differentiation of DRCs. In vitro experiments we verified that *β*‐catenin has a positive regulatory effect on DRCs, suggesting that the Wnt signalling pathway can regulate the differentiation of neonatal DRCs, supporting the significance of Wnt/*β*‐catenin signalling in the repair of bile duct injury. Although the relevance of DRCs for promoting bile duct regeneration, the mechanism regulates this process is not fully understood. Further research is needed to elucidate the more detail of Wnt/*β*‐catenin signalling in DRCs. We encourage more work on these processes, which will hopefully lead to better therapeutic strategies for curing cholangiopathies.

In conclusion, this study not only expands our understanding of the Wnt/*β*‐catenin signalling pathway and its regulatory mechanisms involved in intrahepatic bile duct regeneration through DRCs proliferation, but also provides a new potential research target for hepatobiliary diseases. However, a more comprehensive understanding of this mechanism between DRCs and Wnt/*β*‐catenin signalling in biliary duct regeneration and repair is still unclear. We hope to make further efforts in future work, with a view to bringing more effective treatment for cholangiopathies.

## CONFLICT OF INTEREST

The authors declare no conflict of interest, financial or otherwise.

## AUTHOR CONTRIBUTION

**Nan Wang:** Data curation (lead); Formal analysis (lead); Investigation (supporting); Methodology (supporting); Project administration (supporting). **Rui Kong:** Data curation (supporting); Formal analysis (supporting); Methodology (supporting). **Wei Han:** Investigation (supporting). **Jie Lu:** Conceptualization (lead); Funding acquisition (equal); Project administration (lead).

## Data Availability

The data of this article are available from the corresponding author on reasonable request.
